# The value of umbilical artery blood gas analysis in the rapid diagnosis of fetomaternal hemorrhage

**DOI:** 10.1097/MD.0000000000038249

**Published:** 2024-05-31

**Authors:** Shan Meng, Qiucheng Jia, Huimin Tang, Jiming Chen, Qing Chang

**Affiliations:** a Centre for Reproductive Medicine, First Affiliated Hospital of Army Military Medical University, Chongqing, China; b Department of Obstetrics & Gynecology, The Affiliated Changzhou NO. 2 People’s Hospital of Nanjing Medical University, Changzhou, Jiangsu Province, China; c Department of Obstetrics & Gynecology, First Affiliated Hospital of Army Military Medical University, Chongqing, China.

**Keywords:** Apgar score, blood gas analysis, fetomaternal hemorrhage, neonatal asphyxia, umbilical artery

## Abstract

As a rare obstetric disease, fetomaternal hemorrhage (FMH) often causes severe fetal anemia, edema and even death, easily to be confused with severe neonatal asphyxia. Currently, there are several ways to detect or predict FMH, however, most of them are flawed and time-consuming, as well as unsuitable for rapid diagnosis and timely intervention of FMH. To explore the values of umbilical artery blood gas analysis in the rapid diagnosis of FMH, providing basis for rapid guidance of newborn rescue. Five cases of neonates with FMH from the First Affiliated Hospital of Army Military Medical University (Chongqing Southwest Hospital) from January 2013 to January 2016 were selected as the study group. Another 9 cases of severe asphyxia neonates were chosen into the control group. The difference in Apgar score and umbilical artery blood gas analysis between the 2 groups at birth was compared, and the treatments and clinical outcomes of the 2 groups were analyzed. The PH value of umbilical artery blood gas analysis in the study group was higher than that of the control group, but the difference was not statistically significant (*P* > .05). In the study group, cases with pH value < 7.0 accounted for 0%, whereas the cases with pH < 7.0 accounted for 66.67% in the control group, and the difference between the 2 groups was statistically significant (*P* < .05). Compared with the control group, the arterial oxygen partial pressure (PO_2_), the absolute value of (PCO_2_), lactic acid (lac) and alkali were not significantly different from those of the control group (*P* > .05), while the total hemoglobin (tHb) and hematocrit (Hct) were significantly lower than the control group (*P* < .0001). In the study group, tHb in the umbilical cord blood of 2 newborns with FMH death was significantly lower than 40 g/L. FMH should be highly suspected when there is an expression of severe asphyxia in neonates, indicated by significantly lower tHb levels in umbilical cord blood. It is helpful to improve the neonatal outcome by FMH neonatal resuscitation as soon as possible.

## 1. Introduction

Fetomaternal hemorrhage (FMH) refers to clinical symptoms of different degrees of fetal blood loss, as well as the clinical syndrome of maternal and fetal blood transfusion hemolytic reaction, often occurs during or before delivery when fetal red blood cells through the damaged placental villus gap into the maternal blood circulation.^[1,2]^ As a rare obstetric disease, FMH occurs in various periods of pregnancy such as prenatal or delivery period, causing severe fetal anemia, edema and even death. The etiology and mechanism of FMH is not clear, it is characterized by lack of specific clinical symptoms, occult occurrence, and difficult prenatal diagnosis, making it difficult to early detection for the majority of the cases. As the lack of awareness of FMH in obstetricians, FMH is often confused with severe asphyxia at birth. The perinatal mortality is extremely high when these cases are only treated by conventional neonatal asphyxia recovery. Although there are several methods can be used to detect or predict FMH, such as maternal erythrocyte acid elution staining (Kleihauer-Betke, KB) test, maternal blood alpha-fetoprotein (AFP) level determination, flow cytometry for maternal-fetal red blood cell identification, and absence of fetal free DNA in maternal circulating blood, but these methods have certain shortcomings and often time-consuming, unsuitable for rapid clinical diagnosis and timely intervention.^[3–6]^ Umbilical artery blood gas analysis has been gradually applied in the diagnosis and evaluation of neonatal asphyxia, which can directly reflect the status of acid-base balance in fetus when delivery. Significantly reduced tHb and hematocrit (Hct) levels often suggest the existence of obvious blood loss or anemia in fetus, which has important guiding significance for rapid diagnosis and differential diagnosis of FMH.

How to quickly and easily diagnose FMH by using simple and rapid detection method is of great clinical significance to improve the success rate of rescue and the prognosis of children with FMH. In this study, we analyzed the clinical data of 5 patients with FMH from the First Affiliated Hospital of Army Military Medical University (Chongqing Southwest Hospital), and the 9 cases of severe asphyxia neonates were treated as the control group. The results of umbilical artery blood gas analysis were compared, aimed to provide a simple and effective method for the rapid diagnosis and differential diagnosis of FMH. This would further improve the accuracy of rapid diagnosis of FMH and the success rate of rescue of FMH children.

## 2. Materials and methods

### 2.1. General information

Five cases of neonates who were diagnosed with FMH by the Obstetrics and Gynecology of the First Affiliated Hospital of Army Military Medical University (Chongqing Southwest Hospital) from January 2013 to January 2016 were selected as the study group. 9 cases of severe asphyxia neonates were selected into the control group at the same time. Neonates in the study group and the control group had a clear diagnosis of FMH and severe asphyxia, excluding the cases with unclear diagnosis or interfering factors (such as neonatal hemolytic anemia) for diagnosis. Meanwhile, cases without sufficient clinical data were not included in the statistical analysis. There were no significant difference between the 2 groups in terms of gestational age, body weight and body length (*P* > .05), and the data were comparable (Table 1).

**Table 1:** Comparison of baseline data between the 2 groups.

Groups	N	Gestational age	Birth weight (g)	Body length (cm)
Study group	5	36.80 ± 2.52	2634 ± 715.0	48.20 ± 1.50
Control group	9	35.13 ± 4.17	1942 ± 832.9	42.33 ± 2.16
T value		0.8066	1.559	1.867
*P* value		.4356	.1450	.0865

#### 2.1.1. FMH diagnostic criteria.^[1,7,8]^

Unexplained neonatal anemia (neonatal anemia diagnostic criteria: capillary blood hemoglobin (Hb) ≤ 145g/ L); erythrocyte acid elution experiment (Kleihaue-Bet Test, referred as KB test) found fetal erythrocytes; fetal hemoglobin content determination in maternal blood: fetal hemoglobin content in maternal blood increased > 3% when FMH; or AFP significantly increased in the maternal serum.

#### 2.1.2. The diagnosis of FMH diagnosis in this group was based on the following

Prenatal clinical manifestations of FMH: reduction or disappearance of fetal movement, fetal arrhythmia, etc; fetal heart rate monitoring abnormalities: low fetal heart rate, reduced variability, fetal heart rate is not accelerated when fetal movement, sinusoidal curve or late deceleration waveforms; fetal edema, liver enlargement in part of the FMH patients when tested with ultrasound sound. Clear neonatal anemia (Hb ≤ 145g/ L), excluding other reasons, such as immune hemolytic anemia; other laboratory indicators meet the diagnostic criteria of FMH.

### 2.2. Apgar scoring

Apgar scoring includes skin color, heart rate, breathing, muscle tension and movement, reflection and other aspects. 2 points for each performance, and the total score is 10 points. Apgar scoring was performed within 1 minute after birth. Apgar scoring criteria: 8 to 10 points are considered as normal, 4 to 7 points is considered as mild asphyxia, and 0 to 3 points is considered as severe asphyxia.

### 2.3. Umbilical artery blood gas analysis

The umbilical cord was cut after childbirth. A length of about 15 cm umbilical cord near the newborn was clamped using 2 disinfectant hemostatic forceps before the establishment of normal spontaneous breathing. Cut off the umbilical cord outward of the hemostatic clamp. Immediately extracted 1ml of umbilical artery blood by heparinized umbilical artery blood collection needle (Suzhou Shi Lai Medical Devices Co., Ltd.), and sealed immediately for the following blood gas analysis. The United States GEM premier300 blood gas analyzer was used for testing, and the reference range value was established after statistics of umbilical artery blood gas analysis.

### 2.4. Indicators of blood gas analysis

The indexes of blood gas analysis mainly include PH value, partial pressure of carbon dioxide (PCO_2_), oxygen partial pressure (PO_2_), alkali residue (BE value), absolute value of lactic acid (lac), hemoglobin value (tHb) and Hct.

### 2.5. Statistical methods

Statistical analysis was performed using SPSS 17.0 statistical software (IBM). Measurement data was presented as mean ± standard deviation (x- ± s), using t test; count data was shown in terms of rate (%), using χ^2^ test. *P* < .05 indicates that the difference was statistically significant.

## 3. Results

### 3.1. Comparison of general data between the 2 groups of children

In the study group, the mean time of gestational age was (36.80 ± 2.52) weeks, the mean birth weight was (2634 ± 715.0) g, and the average length was (48.20 ± 1.50) cm. In the control group, the average gestational age was (35.13 ± 4.17) weeks, the mean birth weight was (1942 ± 832.9) g, and the average length was (42.33 ± 2.16) cm. There was no significant difference in general data about gestational age, birth weight and body length between the 2 groups (*P* > .05, Table 1).

### 3.2. Comparison of the Apgar score between the 2 groups

**Figure 1.**
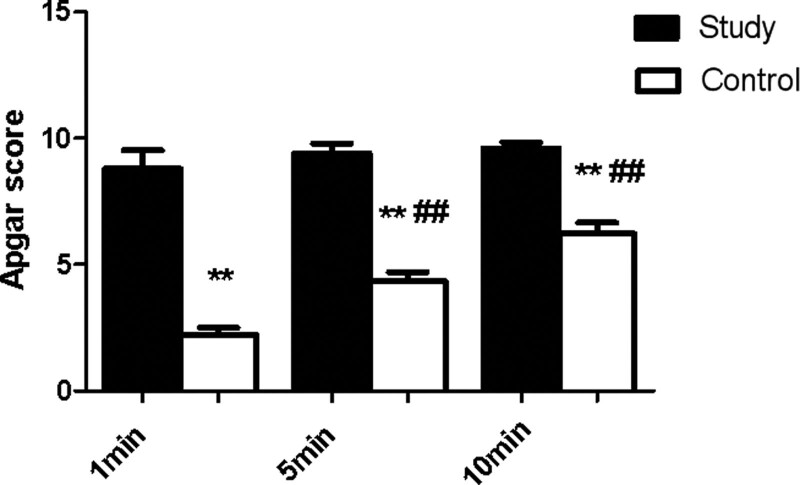
Comparison of Apgar score between newborns in the 2 groups. *P < .05, **P < .01, vs study group at different timeset; #*P* < .05, ##*P* < .01, vs 1 min control.

The Apgar score was 8.80 ± 1.64 versus 2.22 ± 0.833 in the study group and the control group 1 minute after birth, respectively. The Apgar score at 1min in the study group was significantly higher than that in the control group (*P* < .01). The Apgar score at 5 minutes after birth was 9.40 ± 0.89 versus 4.33 ± 1.12 in the study group and the control group, respectively. The Apgar score at 5 minutes in the study group was significantly higher than that in the control group (*P* < .01). The Apgar score at 10 minutes after birth was 9.60 ± 0.55 in the study group and 6.22 ± 1.30 in the control group. The Apgar score at 10 minutes was significantly higher in the study group than that in the control group (Fig. 1).

### 3.3. Comparison of blood gas analysis results between the 2 groups

Compared with the control group, the PH value of the study group was higher than that of the control group (7.23 ± 0.09 vs 6.99 ± 0.25), but the difference was not statistically significant (*P* > .05). However, the results were different when setting various PH cutoff values (Table 2). When PH < 7.2 was used as the cutoff value, there were 1 case in the study group and 6 cases in the control group. There was no significant difference between the 2 groups (1/5 (20%) vs 6/9 (66.67) %), *P* > .05). When PH < 7.0 was used as the cutoff value, there was 0 case in the study group and 6 cases in the control group. The difference between the 2 groups was statistically significant (0/5 (0%) vs 6/9 (66.67 %), *P* < .05). Compared with the control group, the PCO2 in the study group was (41.88 ± 15.92 vs 36.69 ± 9.19 mm Hg), and the difference was not statistically significant (*P* > .05). In term of PO2, there was no significant difference (*P* > .05) between the study group and the control group (50.26 ± 8.28 vs 48.47 ± 3.85 mm Hg). For Lac, there was no significant difference between the study group and the control group (9.78 ± 3.57 vs 10.91 ± 1.45 mm Hg, *P* > .05). For alkali, there was no significant difference between the study group and the control group (−10.72 ± 2.74 vs −11.64 ± 1.61 mm Hg, *P* > .05). For tHb, the level of tHb in the study group was significantly lower than that in the control group (47.20 ± 14.82 vs 143.2 ± 9.36 g/L, *P* < .0001). The percentage of Hct in the study group was significantly lower than that in the control group (15.14 ± 4.77 vs 43.66 ± 2.67%, *P* < .0001).

**Table 2:** Comparison of blood gas analysis results between the 2 groups.

Indicators	Study group (n = 5)	Control group (n = 9)	T value	*P* value
PH value	7.23 ± 0.09	6.99 ± 0.25	2.033	.0648
PCO2 (mm Hg)	41.88 ± 15.92	36.69 ± 9.19	0.7842	.4481
PO2 (mm Hg)	50.26 ± 8.28	48.47 ± 3.85	0. 2256	.8253
Lac (mmol/L)	9.78 ± 3. 57	10.91 ± 1.45	0.3479	.7339
ABE (mmol/L)	−10.72 ± 2.74	−11.64 ± 1.61	0.3130	.7597
tHb (g/L)	47.20 ± 14.82	143.2 ± 9.36	5.764	<.0001
Hct (%)	15.14 ± 4.77	43.66 ± 2.67	5.798	<.0001

ABE = alkali, Hct = hematocrit, lac = lactic acid, PCO2 = pressure of carbon dioxide, PO2 = oxygen partial pressure, tHb = total hemoglobin.

### 3.4. The main treatment measures and clinical outcomes in the 2 groups

The children in the study group were treated with blood transfusion after the routine treatment. The patients in the control group were treated with neonatal asphyxia recovery procedure and transferred to neonatal department for follow-up treatment. In the study group, 2 cases of FMH children died. The levels of tHb in the neonatal umbilical cord blood of these 2 cases of FMH were significantly lower than 40 g/ L (tHb failed to be measured in 1 case, and tHb in another case was 38g/ L). In the control group, 9 cases of asphyxia neonatal, 1 died because of abandoned treatment.

## 4. Discussion

The etiology of FHM is not clear and it is suggested that the causes of FHM may be due to the formation of pressure differences between fetal umbilical artery and villus gap, leading to fetal blood directly enter into the chorionic space and countercurrent into the mother blood circulation. This phenomenon is rarely presented under normal circumstances, mostly in the case of chorionic injury. Because the characteristics of FMH includes low incidence, occult occurrence, and untypical clinical symptoms, as well as it is rarely clinically known, making it difficult to early prenatal detection for the majority of the cases. As a result, FMH often causes serious complications, and perinatal mortality is extremely high.^[9]^

As reported, prenatal clinical manifestations of FMH mainly include reduction or disappearance of fetal movement, fetal arrhythmia, etc. Fetal heart rate monitoring can be expressed as fetal low baseline heartbeat, reduced variability, the fetal heart rate is not accelerated when fetal movement, even sinusoidal or late deceleration waveforms in some typical cases. Fetal edema and liver enlargement can be found by ultrasound test in some FMH cases. But these clinical manifestations are not unique to FMH, and some FMH cases may not have any specific performance.^[10]^ Moreover, the B-ultrasound for early diagnosis of FMH has great limitations.^[11]^ Thus, prenatal diagnosis of FMH is not easy. The appearance of skin in the newborns of FMH usually presents as mucous membrane pale, and this characteristic appearance is easily to be confused with neonatal severe asphyxia (pale suffocation). First aid measures for FMH are significantly different from the neonatal pale asphyxia. The positive routine resuscitation cannot significantly improve the clinical outcome of children with FMH. For FMH, delayed diagnosis and improper treatment measures often cause serious consequences. The retrospectively analysis of Markham LA et al^[12]^ reported that, one case presented as good muscle tone, good vitality with pale skin, 8 and 8 scores of Apgar score at 1, 5 minutes, without timely neonatal diagnosis of acute FMH and timely correction of anemia and low blood volume, the condition of this case was progressive deterioration, and eventually died of multiple organ dysfunction. In view of this, rapid diagnosis of FMH during childbirth, timely identification from neonatal asphyxia, rapid and accurate clinical intervention, is particularly important to improve the success rate of FMH rescue. This study found that: when PH < 7.0 as the cutoff value, there was 0 case of FMH in the study group, while 6 cases of FMH in the control group, and the difference was statistically significant, suggesting that newborns with severe asphyxia may be more likely to suffer from severe acidosis than cases with FMH. Indicators of anemia such as tHb and Hct (%) can be used for better distinguish between FMH and neonatal severe asphyxia, as tHb and Hct levels in cases of FMH are often significantly reduced than normal control.

Although there are several methods can be used to detect or predict FMH, such as maternal erythrocyte acid elution staining (KB) test, maternal blood AFP level determination, flow cytometry for maternal-fetal red blood cell identification, and absence of fetal free DNA in maternal circulating blood, but these methods have certain shortcomings and often time-consuming, unsuitable for rapid clinical diagnosis and timely intervention.^[3–6]^ Umbilical artery blood gas analysis has been gradually applied in the diagnosis and evaluation of neonatal asphyxia, which can directly reflect the status of acid-base balance in fetus when delivery. Significantly reduced tHb and Hct levels often suggest the existence of obvious blood loss or anemia in fetus, which has important guiding significance for rapid diagnosis and differential diagnosis of FMH.

Long-term prognosis of FMH differs between different reports, mainly depends on the amount of bleeding, bleeding speed and body compensatory capacity. 14% of newborns with moderate to severe FMH died.^[13]^ Christensen et al^[14]^ reported the results of multi-center study and pointed out that the incidences of adverse events in terms of death, intraventricular hemorrhage, periventricular white matter softening, bronchial dysplasia, and hypoxic-ischemic encephalopathy were as high as 71% in FMH children with Hct < 30% and hemoglobin < 100g/L at childbirth. In the study group, 2 cases of FMH children died. The levels of tHb in the neonatal umbilical cord blood of these 2 cases of FMH were significantly lower than 40 g/L (tHb failed to be measured in 1 case, and tHb in another case was 38 g/L), suggesting that the level of Hb in umbilical cord blood may be important for predicting the clinical outcome of FMH neonates.

In brief, umbilical arterial blood gas analysis may be of great significance for the early detection of FMH. For neonates after childbirth, it should be highly considered FMH when there are symptoms such as low Apgar score, difficult recovery, abnormal amniotic fluid, slightly lower PH value by the fetal blood analysis, and significantly lower levels of the fetus Hb and Hct (%), especially the neonatal pale and asphyxia degree are inconsistency. FMH once diagnosed, the child pale and the degree of asphyxia is often inconsistent, and conventional neonatal resuscitation is also often ineffective.^[15–17]^ At this time, under the premise of effective vital signs, the newborn should be quickly transferred to NICU for timely fetal blood transfusion or plasma exchange therapy, thereby effectively improving the survival rate of perinatal FMH.^[18–20]^ Rapid and accurate diagnosis of FMH is of great significance to improve the success rate of treatment.

Because FMH is rare, the limitations of this study are retrospective analysis of small sample of clinical cases, non-randomized, and inherent selective bias. In future studies, it should be focused on criteria and cutoff value of umbilical arterial blood gas analysis in rapid diagnosis of FMH. Large samples of clinical trials are needed to further demonstrate the role and value of the umbilical artery blood gas analysis in improving the accuracy of rapid diagnosis of FMH and the success rate of FMH rescue.

Conclusion Umbilical artery blood gas analysis may be contribute to the rapid diagnosis and differential diagnosis of FMH during childbirth, which may be helpful for the rapid rescue of FMH. Neonates with severe asphyxia performance, significantly lower total hemoglobin of umbilical cord blood found by immediate umbilical artery blood gas analysis, should be highly suspected as FMH. Neonatal recovery mode of FMH treatment should be used as soon as possible to improve the neonatal outcome.

## Author contributions

**Conceptualization:** Shan Meng, Qing Chang.

**Data curation:** Shan Meng, Qiucheng Jia, Huimin Tang.

**Formal analysis:** Shan Meng, Qing Chang.

**Funding acquisition:** Huimin Tang.

**Investigation:** Huimin Tang, Qing Chang.

**Resources:** Huimin Tang.

**Visualization:** Jiming Chen.

**Writing – original draft:** Shan Meng, Qiucheng Jia.

**Writing – review & editing:** Qing Chang, Jiming Chen.
